# La mort encéphalique post traumatique: épidémiologie et facteurs de risque

**DOI:** 10.11604/pamj.2018.31.29.16354

**Published:** 2018-09-13

**Authors:** Ahmed Youssef Kada, Kheireddine Abdelouahed Bouyoucef

**Affiliations:** 1 Service de Neurochirurgie, CHU de Blida, Algérie; 2 Université Saad Dahlab, Faculté de Médecine, Blida, Algérie

**Keywords:** Mort, encéphalique, facteur, prédictif, Death, encephalic, factor, predictive

## Abstract

Il est possible de prévoir l'évolution d'un patient vers l'état de mort encéphalique et plusieurs équipes se sont intéressées à la recherche de facteurs prédictifs de la mort encéphalique, cependant la difficulté de proposer un modèle cohérent réside dans le choix des critères proposés, qui, dans la plupart des études est subjectif. Il conviendrait d'analyser une population uniforme et de définir des critères universels pour pouvoir prédire avec certitude le passage en mort encéphalique, un score prédictif étant alors parfaitement envisageable.

## Abstract (en)

Outcome of patients with brain death can be predicted. For this reason, several research teams attempted to identify predictors of brain death. The difficulty of proposing a coherent model lies in the choice of the proposed criteria which, in most studies, are subjective. It would be appropriate to analyze a uniform population and to identify universal criteria in order to predict with certainty brain death and to obtain a feasibility score.

## Introduction

La mort encéphalique a pour origine un arrêt de la circulation cérébrale, parmi les causes possibles de cet arrêt circulatoire, tous les phénomènes qui contribuent à augmenter la pression intracrânienne (PIC), et qui tendent par la suite à diminuer la pression de perfusion cérébrale (PPC). La pression de perfusion intracérébrale (PPC) est la pression nécessaire à la circulation sanguine, le niveau de celle-ci dépend de la pression artérielle moyenne (PAM) et de la pression intracrânienne (PIC), ils sont régis par la formule:

PPC = PAM – PIC

Une augmentation croissante de la PIC non compensée entraine une interruption de la circulation suivie d'une ischémie cérébrale aboutissant secondairement à une destruction irréversible des structures encéphaliques et donc un état de mort encéphalique, celui-ci va se caractériser par la disparition de toutes les fonctions cérébrales. La mort encéphalique est une des conséquences du traumatisme crânien grave, tenter d'éviter cet évènement nécessite une optimisation de la stratégie thérapeutique, le but de cette étude cas-témoins était d'en rechercher les facteurs prédictifs possibles dans le cas particulier de la neurotraumatologie afin de proposer dans un temps raisonnable des alternatives thérapeutiques telle que la réalisation d'un volet décompressif bilatéral [1].

## Méthodes

Nous avons inclus prospectivement tous les patients hospitalisés pour traumatisme crânien grave entre septembre 2008 et septembre 2013, ayant évolués vers la mort encéphalique, le seul critère d'exclusion était la présence d'un facteur confondant. Nous les avons appariés avec des traumatisés crânien décédés, mais de cause non neurologique. La mort encéphalique était définie selon les critères de l'AAN [2] (American Academy of Neurology). Les données cliniques, biologiques et radiologiques ont été recueillies: l'âge, le sexe, le score de Glasgow à l'admission, la classification tomodensitométrique des lésions [3] (Classification de Marshall), la notion de transport inter hospitalier, l'urémie à l'admission. Les données épidémiologiques ont étés recueillies et analysées par le biais des logiciels statistiques gratuit OpenEpi et BiostaTgv. Nous avons réalisé une analyse univariée simple avec calculs manuels puis multivariée par régression logistique utilisant la procédure pas à pas descendant (backward elimination) qui consiste à inclure toutes les variables choisies puis de retirer progressivement les non significatives pour identifier finalement les facteurs prédictifs de mort encéphalique post traumatique les plus pertinents.

## Résultats

Au total, 95 cas et 95 témoins ont été inclus dans notre étude, tous ont bénéficiés d'une prise en charge thérapeutique sans limitation de soins, en raison de la législation algérienne ne prévoyant pas la limitation de l'engagement thérapeutique. Les résultats principaux sont présentés dans le Tableau 1. Le score moyen à l'admission était de 5,40 ± 2,29 chez les cas et de 6,96 ± 3,45 chez les témoins (p=0,01), L'âge moyen étant de 25,88 ± 15,08 ans chez les cas et de 38,71 ± 21,53 ans chez les témoins (p=0,01). Après analyse statistique (Tableau 2), le sexe masculin (p=0,004; OR=3), la notion de transport >60mn (p=0,02; OR=2), l'âge <50 ans (p=0,0001; OR=6), une classe IV sur la classification scannographique de Marshall (p=0,01; OR=5,47) et une urémie élevée (p=0,01; OR=5,7) étaient retrouvés comme prédictifs de mort encéphalique en analyse univariée. En analyse multivariée, les facteurs prédictifs de cet événement étaient l'âge inférieur à 50 ans (p=0,000; OR=8,9) et la présence d'une mydriase unilatérale (p=0,025; OR=4,026).

**Tableau 1:** Résultats

	Cas	Témoins	p
**Sexe ratio (H/F)**	85/10	70/25	<0.01
**Score de Glasgow**	5,40±2,29	6,96 ± 3,45	<0.01
**Age moyen (ans)**	25,88±15,08	38,71 ± 21,53	<0.01
**Urée plasmatique (mmol/L)**	6,99±4,82	4,82±1,66	<0.01
**Glycémie (mmol/L)**	8,91±2,31	8,85±2,42	ns
**TP (%)**	70,39±18,76	70,77±29,07	ns
**Hémoglobine (g/dl)**	12,18±1,32	11,93±1,80	ns
**Délai avant admission (heures)**	7,39±2,79	7,92±8,16	ns
**Survie moyenne (heures)**	130,66±10,77	134,22±13,71	ns

**Tableau 2:** Analyse statistique

	Analyse univariée			Analyse multivariée		
Variable	OR	IC à 95%	p	OR	IC à 95%	p
**Sexe**	3	1,36-6,74	p=0,004			
**Transport**	2	1,05-3,92	p=0,024			
**Age<50**	6	2,42-15,82	p=0,000	8,9	3,07-25,74	p=0,000
**Glasgow<7**	1,6	0,90-2,84	p=0,072			
**TDM**	5	1,16-25,68	p=0,016			
**Mydriase unil**	1,8	0,68-4,83	p=0,167	4,026	1,18-13,63	p=0,025
**Urée>0,50g/l**	5,7	1,07-30,72	P=0,034			

## Discussion

Il nous a été possible de mettre en évidence des facteurs prédictifs de l'évolution d'un patient vers l'état de mort encéphalique. Dans les limites de notre étude, deux facteurs de risque sont ressortis de manière significative, il s'agit du facteur âge (un âge inférieur à 50 ans multiplie par 9 le risque d'évoluer vers la mort encéphalique lors d'un traumatisme crânien grave) et de la présence d'une mydriase unilatérale (la présence d'une mydriase unilatérale, signe d'un engagement cérébral imminent multiplie par 4 le risque d'évoluer vers la mort encéphalique lors d'un traumatisme crânien grave). Ces éléments permettront d'anticiper une évolution défavorable et d'adapter le protocole thérapeutique en conséquence, en particulier la réalisation d'un volet décompressif lorsque les paliers thérapeutique sont atteint, et inefficaces. En effet les indications des volets decompressifs ne sont pas posées avec certitude et le cas par cas et de règle à l'heure actuelle [4,5], il est cependant certain que le volet bilatéral peut apporter une solution thérapeutique dans les cas extrêmes. Plusieurs équipes se sont intéressés à la recherche de facteurs prédictifs de la mort encéphalique, certaines ont pu en définir quelques-uns, tel que le volume d'hématome [6] ou l'hyperglycémie [7], d'autres non [8]. La difficulté de proposer un modèle prédictif réside dans le choix des critères proposés, dans la plupart des études il est subjectif, et dépend de la population analysée et des prédicteurs choisis, il conviendrait d'analyser une population uniforme et de définir des critères universels pour pouvoir prédire avec certitude le passage en mort encéphalique.

## Conclusion

La mise en évidence de facteurs de passage vers la mort en céphalique est importante dans la mesure où elle pourrait permettre l'utilisation d'une technique de sauvetage non encore admise dans la pratique courante à l'instar de la réalisation des volets decompressifs [1].

### Etat des connaissances actuelles sur le sujet

- Pathologie rare, la mort encéphalique est souvent imprévisible, seule la gravité du traumatisme initial peut donner une estimation du pronostic;
- Les études concernant les facteurs prédictifs de survenue de mort encéphalique sont rares, les auteurs se concentrant souvent sur le traumatisme crânien grave;
- La pénurie d'organes étant un problème majeur pour les équipes de transplantation, prévoir la survenue d'un état de mort encéphalique pourrait permettre une évolution des stratégies ou des protocoles de transplantation.

### Contribution de notre étude à la connaissance

- Dans les limites de cette étude, nous rapportons la présence de facteur prédictifs de mort encéphalique;
- Ces facteurs de risques pourraient permettre une anticipation de la survenue de la mort encéphalique et la mise en route de solutions thérapeutiques extrêmes telles que la réalisation de volets decompressifs bilatéraux;
- Cette étude ouvre la possibilité d'élaboration d'un score prédictif de mort encéphalique en neurotraumatologie.

## Conflits d’intérêts

Les auteurs ne déclarent aucun conflit d'intérêts.
